# Towards Fully Integrated Wireless Impedimetric Sensors

**DOI:** 10.3390/s100404071

**Published:** 2010-04-21

**Authors:** Fredy Segura-Quijano, Jordi Sacristán-Riquelme, Jesús García-Cantón, Maria Teresa Osés, Antonio Baldi

**Affiliations:** Instituto de Microelectrónica de Barcelona, IMB-CNM (CSIC), Campus UAB, E-08193 Cerdanyola, Spain; E-Mails: fsegura@uniandes.edu.co (F.S.-Q.); Jordi.sacristan@imb-cnm.csic.es (J.S.-R.); jesus.garcia@imb-cnm.csic.es (J.G.-C.)

**Keywords:** wireless sensors, telemetry, impedimetric sensors, CMOS sensors

## Abstract

We report on the design and characterization of the building blocks of a single-chip wireless chemical sensor fabricated with a commercial complementary metal-oxide-silicon (CMOS) technology, which includes two types of transducers for impedimetric measurements (4-electrode array and two interdigitated electrodes), instrumentation circuits, and a metal coil and circuits for inductive power and data transfer. The electrodes have been formed with a polycrystalline silicon layer of the technology by a simple post-process that does not require additional deposition or lithography steps, but just etching steps. A linear response to both conductivity and permittivity of solutions has been obtained. Wireless communication of the sensor chip with a readout unit has been demonstrated. The design of the chip was prepared for individual block characterization and not for full system characterization. The integration of chemical transducers within monolithic wireless platforms will lead to smaller, cheaper, and more reliable chemical microsensors, and will open up the door to numerous new applications where liquid mediums that are enclosed in sealed receptacles have to be measured.

## 1. Introduction

The combination of wireless readout and single-chip integration features is particularly attractive for chemical and biochemical sensors fabricated using microtechnologies. Such single-chip systems will not require wire-bond or any other type of electrical connection to external components or circuits, thereby enabling full packaging at the wafer level. Packaging has always been one of the most critical aspects of chemical microsensors due to the difficulty in reliably protecting their electrical connections while exposing part of the chip to the liquid environments they are meant to measure. Hence, avoiding such electrical connections should be very advantageous in terms of cost and reliability. On the other hand, their small size and wireless readout capability should be very convenient for many applications requiring the measurement of liquid mediums enclosed in sealed receptacles. Single-chip wireless chemical sensors based on passive resonant structures have been reported in the past [1,2]. These sensors can be suitable for the specific applications they have been designed for. However, a higher design flexibility and sensor smartness can be achieved using standard microelectronic fabrication technologies, e.g., complementary metal-oxide-silicon (CMOS), for their implementation. Measurement and communication circuits are readily implemented with CMOS technologies, but integration of the chemical transducers and the power and data transfer antenna is more challenging. Different strategies have been used for the integration of these components. For example, electrodes for amperometric [3] and impedimetric [4] sensors have been typically integrated by noble metal deposition and lift-off process. High quality factor coils for power and data transmission in monolithic wireless systems have been integrated by electrodeposition of a thick copper layer in a photoresist mould and subsequent seed-layer etching [5,6]. However, the more complex the post-process of an integrated system is, the more difficult and expensive to mass produce it.

Here we present the design, fabrication and characterization of a single chip wireless chemical sensor based on a commercial 0.35 μm CMOS technology. The focus of our approach has been to keep the post-processing simple, with no additional deposition or lithography steps. The chip includes two types of impedimetric transducers, a 4-electrode array and a pair of interdigitated electrodes, for the implementation of chemical sensors. Impedimetric transducers are easy to integrate because they do not require a reference electrode, unlike potentiometric or amperometric ones. By measurement of changes in conductivity or permittivity of a solution, this transducers have been used to acquire chemical parameters, like pCO_2_ [7], ethanol content in fuel [8], and hematocrit [9]. Biosensors have been obtained by combining impedimetric transducers with enzymes [10–12], and antibodies [13,14]. They have also been combined with selective membranes for the detection of ions such us calcium [15] and pH [16]. Therefore, the integration of impedimetric transducers with their associated instrumentation, inductive powering, and data transmission circuits will enable the development of a wide variety of single-chip, wafer-level encapsulated wireless chemical sensors.

## 2. Design and Fabrication

**Figure 1.**
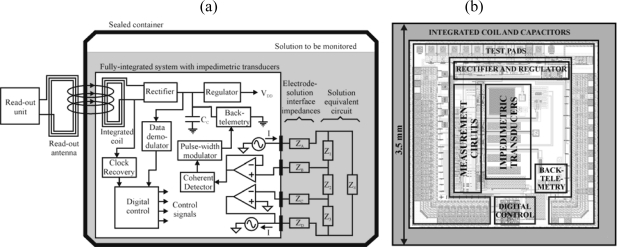
(a) Block diagram of the wireless chemical sensor measuring in the solution of a sealed container. (b) Distribution of blocks and components on the actual chip design (represented as the superposition of fabrication masks).

The block diagram of the system is depicted in Figure 1a. The external readout unit transfers power and control data to the chip by inductive telemetry, that is, by generating an alternating magnetic field with a loop antenna, which in turn produces an alternating electromotive force at the integrated coil. The received voltage is rectified and regulated to generate a stable voltage supply for the integrated circuits. The impedimetric transducers are excited and measured with analog signal processing circuits, and the resulting impedance value is converted to the digital domain with a simple Pulse Width Modulation technique and transmitted back to the readout unit by load modulation. The process of measurement and data transmission is controlled by a digital block. The data to program the digital block is transferred from the external readout unit by amplitude modulation of the power signal.

The 3.5 × 3.5 mm chip (Figure 1b) containing all the blocks and components shown in Figure 1a diagram was fabricated with a commercial CMOS technology (AMS035HV4M, Austria Microsystems) and post-processed in our laboratory. Various blocks and components of the system were made accessible through connection pads to allow their individual characterization.

### 2.1. Impedimetric transducers

The electrodes for the impedimetric transducers have been formed in polycrystalline silicon (polysilicon). One or two layers of this material are typically used in CMOS technologies for the transistor gate and capacitor electrodes. Polysilicon has the advantage over the metal interconnection layers (made of aluminum or copper) that does not degrade in contact with aqueous solution. A passivating thin layer (∼3 nm) of native oxide grows spontaneously and stops further corrosion of the material. This thin layer of native oxide prevents using faradic processes for chemical detection. However it does not prevent their use in measuring conductivity and permittivity of the media between electrodes [11,17]. The impedance of the electrode-solution interface in this type of electrodes has been shown to behave as constant phase element [11]:

(1) $$Z_{\text{int}}=\frac{1}{C_{\mathrm{CPE}}{\left(j\omega \right)}^{\alpha_{\mathrm{CPE}}}}$$

The modulus of this impedance decreases with increasing frequency, so that above a given frequency its value is small compared to the impedances associated with the solution.

As already mentioned, two types of impedimetric transducers are tested: a 4-electrode array and a pair of interdigitated electrodes. The 4-electrode design is suitable for precise conductometric measurements in a wide range of conductivities. Injection of current through the external electrodes and measurement of voltage across the internal ones allows measurement of the conductance without interference of the interface impedance. However, due to a much higher capacitance through the silicon substrate than through the solution, this 4-electrode transducer is not suitable for measurement of permittivity. On the other hand, interdigitated electrodes can measure both conductivity and permittivity. In this case the impedance of the electrodes is in series with the impedance being measured, and thus, they are not suitable for measurements of very high conductivity media, which yields lower impedance than the electrodes themselves. Due to the short penetration depth of the currents generated by these electrodes, they are especially suited for the measurement of thin membranes and phenomena occurring close to the surface [13,14].

**Figure 2.**
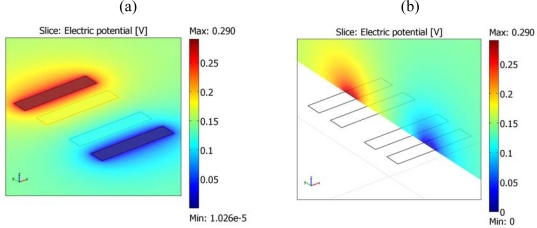
Distribution of electric potential in the 4-electrode array for a current of 10 μA through the external electrodes and a 1 mS/cm solution at the surface (a) and at a cross-section (b).

The area of the electrodes for both 4-electrode and interdigitated designs was chosen large enough so as to allow measurement of their impedance in a wide range of solution conductivities with reasonable values of injected currents and measured voltages within the 3.3 V of the voltage supply. Finite Element Analysis (FEA) simulations (Comsol Multiphysics™) were used to estimate the values of the impedance for different geometries and solution parameters. Each electrode of the 4-electrode array is a 500 × 100 μm^2^ rectangle. Distance between external and internal electrodes is 100 μm, and between internal electrodes is 200 μm. The interdigitated electrodes consist of 70 fingers spaced 3 μm, each having a length of 400 μm and a width of 3 μm. Figure 2 shows the distribution of electric potential at the 4-electrode array when a current of 10 μA is injected through one external electrode and collected at the other external electrode in a solution with conductivity of 1 mS/cm. The peak-to-peak voltage in this case is 580 mV, which is compatible with the available voltage supply. However, for a 100 μS/cm solution the peak-to-peak voltage would be 5.8 V, and hence a smaller excitation current would be necessary (e.g., 1 μA). The total resistance between external electrodes is 29 kΩ for the 1 mS/cm. The transresistance of the array, defined as:

(2) $$r_{m}\equiv V_{i}/I_{e}$$

where V_i_ is the voltage amplitude at the internal electrodes and I_e_ is the current amplitude at the external electrodes, is 6.2 kΩ. This yields a cell constant of 6.2 cm^−1^. The resistance between the interdigitated electrodes for a 1 mS/cm aqueous solution is 1.61 kΩ as calculated from the simulations, which results in a cell constant of 1.61 cm^−1^. This cell constant can also be used to estimate the sensitivity of the electrodes to changes in permittivity [11]:

(3) k≡R/ρ=ε/C

where R is the measured resistance, ρ is the resistivity, ε is the permittivity and C is the measured capacitance.

**Figure 3.**
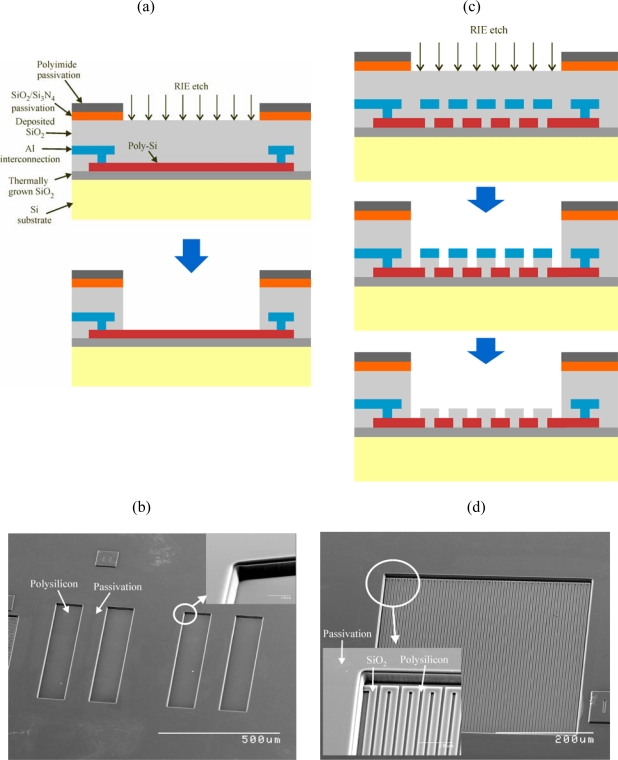
Post-process of the CMOS chip: (a) A simple RIE etch is used to expose the large polysilicon electrodes. (b) SEM image of the 4-electrode array. (c) To expose the interdigitated electrodes, a metal layer is used to protect the silicon oxide between fingers during the RIE etch. (d) SEM image of the interdigitated electrodes.

The post-process steps are performed at the chip level. However, wafer-level processing would be identical. The post-process to form the 4-electrode array in the CMOS chip consists on just one plasma etch (Figure 3a). The electrodes are defined in the second polysilicon layer of the technology (the one used in polysilicon-polysilicon capacitors). The area of the electrode to be exposed to the solution is defined in the pad layer. This layer indicates the areas of passivation to be etched and is typically used to open the metal area of the connection pads. Since there is no metal on top of the polysilicon electrodes, the etching of the passivation during the CMOS fabrication will also etch some of the underlying intermetal dielectric layers. The remaining silicon oxide layers (total thickness ∼5 μm) are removed in the post-process with a selective CHF_3_-based Reactive Ion Etch (RIE) step. The passivation in the AMS technology used includes a 4.5 μm-thick layer of polyimide that protect the rest of the chip during the RIE etch. Figure 3b show a scanning electron microscope (SEM) image of the 4-electrode array after post-processing.

The post-process to form the interdigitated electrodes is slightly different. The area of the interdigitated electrodes to be exposed to the solution is again defined in the pad layer. In this case, a line of the first metal layer has been included between the polysilicon fingers as an etch-stop to avoid exposing the silicon substrate by over-etching of the SiO_2_ layers. Once the SiO_2_ on top of the electrodes has been removed with the RIE step, an additional etch is necessary to remove the metal line (Figure 2c). During the metal etch, carried out in “piranha” solution (70% (v/v) H_2_SO_4_, 30% (v/v) H_2_O_2_) for 5 min, the connection pad areas are protected with a teflon piece pressed against the chip. It is important to note that the present prototype has connection pads for the individual test of the different blocks, but a real design would not need any pads, and hence, would not have any other metal area exposed to this etch. Figure 2d shows a SEM image of the interdigitated electrodes after post-processing.

### 2.2. Measurement circuit

The block diagram of the measurement circuit is included in Figure 1a. The details of the circuit design are described elsewhere [18]. In brief, two sinusoidal current generators with 180° phase difference inject a current through the external electrodes, which produces a voltage across the internal electrodes with amplitude proportional to the impedance. The real and imaginary components of the impedance are extracted by coherent detection using a reference signal in phase and in quadrature with the injected current, respectively. The symmetric injection of currents through the external electrodes allows maintaining one internal electrode at a virtual ground and measuring a DC free differential voltage at the other internal electrode, thus avoiding the use of large capacitance filters that would be difficult to integrate. The sinusoidal current generators are implemented with a direct digital synthesis technique, which is low-power and allows fine tuning of the phase. The circuit can be used with both the 4-electrode array and the interdigitated electrodes. When measuring interdigitated electrodes each electrode is connected to one current generator and one voltage sensing input. The digital block can be externally programmed to run the measurement with different parameters, including 4 current values (0.5 μA, 1 μA, 5 μA and 10 μA) yielding different measurement ranges, phase error compensation with 0.3° resolution, frequency selection in the 10–100 kHz range, and selection of the real or imaginary component.

### 2.3. Inductive telemetry

Power and data for the integrated system are transferred wirelessly by classical telemetry at 13.56 MHz, which is in an Industrial, Scientific, and Medical (ISM) radio band. This frequency is suitable for transmission through aqueous solutions without significant losses by induced eddy currents. In the present system the receiver coil is monolithically integrated, and therefore the readout distance is limited to a few millimeters [19]. The coil is implemented with the metal layers of the CMOS technology, which avoids the requirement of additional post-process but limits the quality factor to about 1. With such quality factor it is not possible to take advantage of the resonance phenomena, and therefore, the coil is directly connected to the rectifier without tuning capacitor. The number of turns was maximized to obtain maximum voltage from the alternating magnetic field, while keeping self-resonant frequency higher than the working frequency. The coil was implemented using three of the metal layers available in the CMOS technology, resulting in an equivalent thickness of 4.1 μm. The coil is a square spiral having 32 turns, internal diameter of 3 mm and external diameter of 3.5 mm, yielding a theoretical inductance and resistance of 7.68 μH and 480 Ω, respectively. Underneath the coil, a large (2.3 nF) poly-poly capacitor for energy storage at the regulator output was implemented.

The readout coil is implemented as a square copper spiral in a printed circuit board, having 9 turns, external diameter of 20 mm, and internal diameter of 2 mm. A load modulation technique is used for the transmission of data from the sensor chip to the readout unit. The analog impedance values are converted to the digital domain with a simple pulse width modulation (PWM) technique.

## 3. Results and Discussion

**Figure 4.**
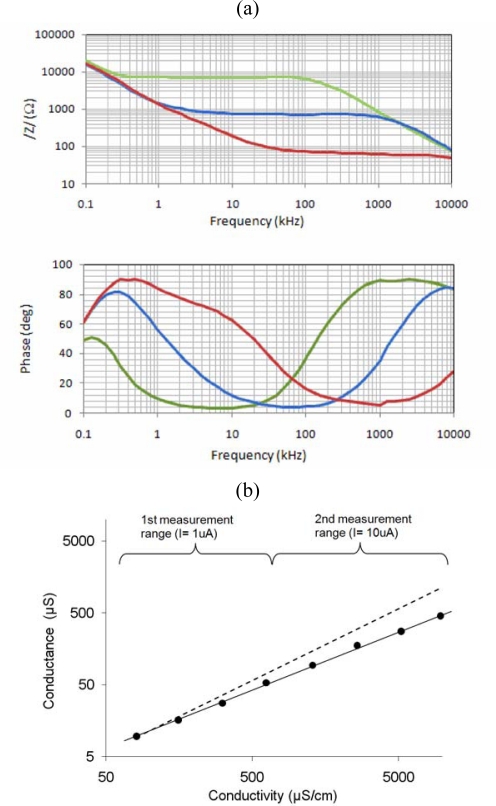
Characterization results of the 4-electrode array: (a) Impedance spectra for 0.97 mS/cm (green), 9.10 mS/cm (blue) and 96.7 mS/cm (red) as measured with an impedance analyzer. (b) Conductance at the electrodes for different solution conductivities measured with the integrated circuit using two different excitation currents (circles) and calculated response from simulations (dashed line).

Figure 4a shows the impedance spectra measured with the 4-electrode array connected to the impedance analyzer in aqueous solutions of different conductivity. The Bode plot shows a wide constant modulus region of approximately two frequency decades with phase close to 0°, which is associated to the resistivity of the solution. Figure 4b shows the real part of the impedance measured with the 4-electrode array at 13.0 kHz using the integrated circuit for the impedance measurement.

Two different measurement ranges (i.e. excitation currents) were used, so that a total measurement range of two orders of magnitude could be achieved. The conductance shown in Figure 4b was calculated as the ratio of measured voltage to excitation current, which is the inverse of the transresistance defined in Equation (2). The estimated response calculated by finite element simulations is also shown in the figure. The slight disagreement with the measured values is probably related to some degree of attenuation at the filters of the integrated coherent detection circuit.

**Figure 5.**
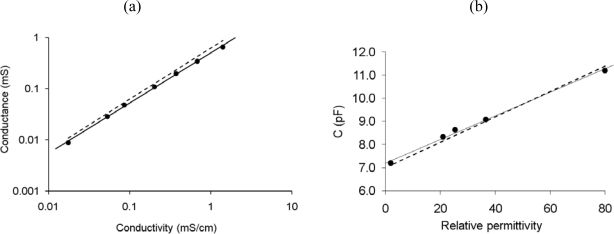
Characterization results of the interdigitated electrodes: (a) Measured conductance for different solution conductivities. (b) Measured capacitance for different solution permittivities. Dashed lines show the response estimated from FEA simulations.

The interdigitated electrodes exhibit a linear response to conductivity and permittivity as shown in Figures 5a and 5b, respectively. The response is obtained from the corresponding components of the equivalent circuit fitted to the impedance spectra as described elsewhere [11]. The linear response to permittivity does not cross the origin due to the parasitic capacitance of the electrodes with the silicon substrate. The cell constant from conductivity measurements is 1.72 cm^−1^ and from permittivity measurements is 1.76 cm^−1^, in good agreement with the values calculated from simulations (1.61 cm^−1^).

Until recently, polysilicon had not been considered as a suitable material for electrodes in electrochemical sensors due to its rapid oxidation in wet environments. However, in impedimetric measurements of the electric properties of solutions, which are not affected by the electrode-solution interface phenomena, the use of polysilicon electrodes has been demonstrated to be suitable for many applications [11,13,14,17]. Here, for the first time, such electrodes have been fabricated with a commercial CMOS technology, and its suitability for impedimetric measurements has been confirmed with the results presented above.

The different components and blocks involved in the wireless transfer of power and data to and from the sensor chip were also tested individually. The integrated coil parameters were measured with the impedance analyzer, yielding an inductance of 6.81 μH, a series resistance of 628 Ω, and a corresponding Q of 1.12, in good agreement with the calculated values. With the readout coil at a distance of 3 mm, the integrated coil received a power of 5 mW. This power is sufficient to supply the system during the programming, measuring, and back telemetry stages. The transfer of measurement parameters was performed by amplitude shift keying (ASK) modulation with a class E amplifier controlled by a field programmable gate array (FPGA). The measurement parameters were successfully transferred to the chip.

At the same distance (3 mm) a load modulation test was performed to validate the back telemetry system. A modulation index of 3.5% was achieved, which is more than necessary for a correct data reception.

## 4. Experimental Section

The different components of the wireless sensor were characterized separately by attaching the chip to a printed circuit board (PCB) and connecting the test pads with wire-bonds. The pads and wires were protected with Epoteck H77 epoxy resin. In this way, the impedimetric transducers could be tested while immersed in different solutions. Solutions with conductivities in the range from 0.1 mS/cm to 10 mS/cm were prepared by diluting a concentrated KCl solution. The conductivity was measured with a CRISON μCM 2202 conductimeter. Solvents with different permittivity, including water (ε_r_ = 80.1), acetonitrile (ε_r_ = 36.6), ethanol (ε_r_ = 25.3), isopropanol (ε_r_ = 21.0) and hexane (ε_r_ = 1.89), were used to test the response of the interdigitated electrodes to permittivity. All chemicals are of reagent grade. The integrated electrodes were measured both with a commercial impedance analyzer (SI 1260 SOLARTRON) using an alternating excitation voltage amplitude of 10 mV, and with the integrated impedance measurement circuit included in the same chip using the predefined excitation currents mentioned in the previous section.

## 5. Conclusions

The results presented here demonstrate that polysilicon impedimetric transducers can be fabricated with a commercial CMOS technology and they yield linear responses to variations of conductivity and permittivity. By adding proper recognition components, implementation of single-chip chemical sensors and biosensors is possible. Integration of the transducers with circuits and components for wireless powering and data transfer has also been demonstrated. The maximum working distance for the present prototype is 3 mm, which is enough for many “through the wall” measurements. A distance in the order of centimeters could be achieved by adding an external independent resonator [20], which would open up the door to other applications like “lab-on-a-pill” devices for gastric disease monitoring [21], and small animal testing with implantable sensors [22]. The present chip design was intended to demonstrate the feasibility of the transducer integration and the wireless readout by individual characterization of the different blocks and components necessary to form the system. The characterization results show a proper functioning of each individual block. In the future, a fully interconnected version of the wireless chemical sensor chip will be designed and its use in real applications demonstrated.
